# Extramammary Paget disease shows differential expression of B7 family members B7-H3, B7-H4, PD-L1, PD-L2 and cancer/testis antigens NY-ESO-1 and MAGE-A

**DOI:** 10.18632/oncotarget.27247

**Published:** 2019-10-22

**Authors:** Maryam Pourmaleki, Jonathan H. Young, Nicholas D. Socci, Sarah Chiang, Marcia Edelweiss, Yanyun Li, Mianlei Zhang, Lev Roshal, Dennis S. Chi, Klaus J. Busam, Ingo K. Mellinghoff, Travis J. Hollmann

**Affiliations:** ^1^ Human Oncology and Pathogenesis Program, Memorial Sloan Kettering Cancer Center, New York, NY 10065, USA; ^2^ Pathology, Memorial Sloan Kettering Cancer Center, New York, NY 10065, USA; ^3^ Bioinformatics Core, Memorial Sloan Kettering Cancer Center, New York, NY 10065, USA; ^4^ Surgery, Memorial Sloan Kettering Cancer Center, New York, NY 10065, USA; ^5^ Neurology, Memorial Sloan Kettering Cancer Center, New York, NY 10065, USA; ^*^ Present address: School of Medicine, Texas Tech University Health Sciences Center, Lubbock, TX 79430, USA

**Keywords:** extramammary paget disease, B7 family, cancer/testis antigens, targetable molecules, immunotherapy

## Abstract

Extramammary Paget disease (EMPD) is a rare cutaneous adenocarcinoma of the anogenital region most commonly treated with surgical excision. Surgical margin clearance is often problematic and recurrence rates remain high indicating the need for additional therapeutic options. Topical immunomodulators have been used with reported success suggesting EMPD may respond to other immunotherapies. This study investigates EMPD protein expression of targetable B7 family members and cancer/testis antigens (CTAs) B7-H3, B7-H4, PD-L1, PD-L2, MAGE-A, and NY-ESO-1 and components of antigen presenting machinery B2M and MHC-I. Fifty-seven specimens from 48 patients (31 female and 17 male), representing *in situ*, invasive, and metastatic disease of primary and secondary origin were stained and scored (627 total slides). The percentage of cases expressing each immune regulatory molecule in the *in situ* followed by invasive tumor components was: B7-H3 (94, 90), B7-H4 (82, 78), PD-L1 (6, 10), MAGE-A (39, 50), NY-ESO-1 (16, 20), B2M (100, 89), and MHC-I (78, 79). PD-L2 was negative in all cases. There was high correlation between marker expression within the *in situ* and invasive tumor components of the same case. B7-H4 was preferentially expressed in primary cutaneous EMPD. Co-expression of B7 family members B7-H3 and B7-H4 was found within the *in situ* and invasive tumor components of 74% and 48% of cases, respectively. These findings provide an initial characterization of EMPD tumor cell expression of B7-H3, B7-H4, PD-L1, PD-L2, MAGE-A, and NY-ESO-1 and indicate the potential for new immunotherapeutic options for patients with EMPD.

## INTRODUCTION

Extramammary Paget disease (EMPD) is a rare cutaneous adenocarcinoma arising most commonly in the anogenital region and rarely in other apocrine-rich areas such as the axilla outside of the mammary gland. Because EMPD can occur in both apocrine-rich and apocrine-poor areas, the cell of origin of EMPD remains controversial. Overall, the disease is most common in Asians with 10 cases per million reported compared to 7 cases per million in Europeans and 0.9 cases per million in Westerners with a slight female predominance. Patients are generally over the age of 65 years [1, 2]. Of European females with EMPD, the majority of disease (83%) is located in the vulva with most studies reporting invasion in 16–23% of cases [3–7]. Invasive EMPD is associated with lymph node or soft tissue metastasis in 34–61% of cases with a post-metastasis 5-year survival of less than 10% [5–10].

Surgical removal of tumor with wide margins is currently the treatment of choice for localized EMPD, though it is often difficult to achieve complete excision with sufficient clearance. Recurrence rates for vulvar Paget disease after surgical treatment range from 34–56% [6, 11–13]. It is likely that numerous factors including anatomic location, depth of adnexal extension, discontinuous areas of tumor growth, and ill-defined tumor margins result in an increased risk of recurrence. Nonsurgical treatments for recurrent disease most commonly include radiation therapy, imiquimod, and photodynamic therapy. While nonsurgical therapies report high response rates, only a minority of patients experience long-term remission [7]. Treatment of metastatic disease is limited with no standard regimen of chemotherapy. Since 20–60% of EMPD cases show *HER2* gene amplification and/or overexpression of the HER2 protein, trastuzumab has been used as monotherapy or in combination with chemotherapy [14–22].

Following the success of first-generation checkpoint inhibitors for cancer treatment, expression of the B7 family member programmed death-ligand 1 (PD-L1) has been widely interrogated in tumors and within the tumor microenvironment. However, not all patients show de-novo or durable response to anti-PD-L1 or anti-PD-1 therapy. Therefore, attention has shifted to other immune checkpoints such as alternative B7 family members or use of combinatorial clinical regimens including checkpoint blockade in combination with cancer/testis antigen (CTA) vaccines or other immunomodulators [23]. The B7 family of immunoregulatory molecules currently consists of 10 members including B7-1 (CD80), B7-2 (CD86), B7-H1 (PD-L1, CD279), B7-DC (PD-L2, CD272), B7-H2 (ICOSL, CD275), B7-H3 (CD276), B7-H4, B7-H5 (VISTA), B7-H6, and B7-H7 (HHLA2). The CTAs currently consist of over 200 molecules including New York esophageal squamous cell carcinoma 1 (NY-ESO-1) and melanoma-associated antigen A (MAGE-A) [24].

Data from ongoing trials suggests that checkpoint blockade may be useful for treatment of non-melanoma skin cancers [25, 26], and other studies have interrogated the use of checkpoint blockade in combination with CTA vaccines [23]. Given this evidence and the clinical need to expand therapeutic options for local and metastatic EMPD, the purpose of this study was to characterize EMPD tumor cell expression of select currently targetable B7 family members, B7-H3, B7-H4, PD-L1, programmed death-ligand 2 (PD-L2) and CTAs, NY-ESO-1 and MAGE-A. Additionally, since response to checkpoint blockade has been associated with expression of major histocompatibility complex class I (MHC-I) and beta-2-microglobulin (B2M) [27], we evaluated EMPD tumor cell expression of MHC-I and B2M in our cohort. To further interrogate the primary versus secondary site of origin in each case and the correlation between expression of the B7 family members and CTAs with cytokeratin 7 (CK7) and cytokeratin 20 (CK20) expression, we evaluated expression of CK7 and CK20 in each case. Lastly, CD8 density was quantified to explore the correlation between CD8 density and expression of all markers. We then performed hierarchical clustering of tumor cell immunohistochemical (IHC) expression scores and correlated these findings with multiple characteristics of the tumor including sex, extent of disease, origin, anatomic site, prior treatment, CK7/CK20 status, vital status, and CD8 density within a series of predominantly *in situ* and invasive EMPD. We also report the incidence of coexpression of the targetable molecules.

## RESULTS

### Patient characteristics

This study included 48 patients with EMPD, 31 of whom were females (64.6%) and 17 males (35.4%), ranging from 39 to 92 years with a median age of 69.5 years (Table 1). In total, 57 cases were characterized, as six patients had two resections and three cases had two tumor blocks. Thirty-three (61.1%) cases represented *in situ* disease, 19 (35.2%) represented invasive disease, and 2 (3.7%) represented metastatic disease. Most samples were primary cutaneous tumors originating in skin (48, 88.9%), while 6 (11.1%) were secondary to cutaneous involvement by a colorectal primary tumor. Charts were reviewed for history of colon cancer to distinguish between primary and secondary disease. Twenty-five (46.3%) cases involved the vulva, and the remaining represented penile/scrotal, perianal, perineal, and other (lymph node, skin/abdomen, buttock, thigh) EMPD. Prior therapies were determined for each patient and noted to account for any differences in the expression of the studied markers. Thirty-seven (68.5%) patients had no prior therapy. Of the 48 patients, 5 (10.4%) died from EMPD with a median disease duration of 73 months ranging from 15–199 months.

**Table 1:** Patient characteristics

	*n*	**%**
**Median age (range)**	69.5 (39-92)	
**Sex**		
Female (biopsies)	31 (33)	64.6
Male (biopsies)	17 (21)	35.4
**Extent of disease**		
*In situ*	33	61.1
Invasive	19	35.2
Metastatic	2	3.7
**Origin**		
Primary	48	88.9
Secondary	6	11.1
**Site**		
Vulvar	25	46.3
Penile/scrotal	9	16.7
Perianal	11	20.4
Perineum	5	9.3
Other	4	7.4
**Medicinal treatment**		
Aldara	7	13.0
Aldara/chemotherapy	1	1.9
Aldara/neratinib	2	3.7
Chemotherapy	3	5.6
5-FU	1	1.9
Other	3	5.6
None	37	68.5
**Vital status**		
Alive	20	41.7
Death from disease	5	10.4
Death unrelated to disease	2	4.2
Unknown	21	43.8

Abbreviation: 5-FU, Fluorouracil.

### Expression of B7 family molecules, cancer/testis antigens, and antigen presenting machinery in EMPD

**Figure 1:**
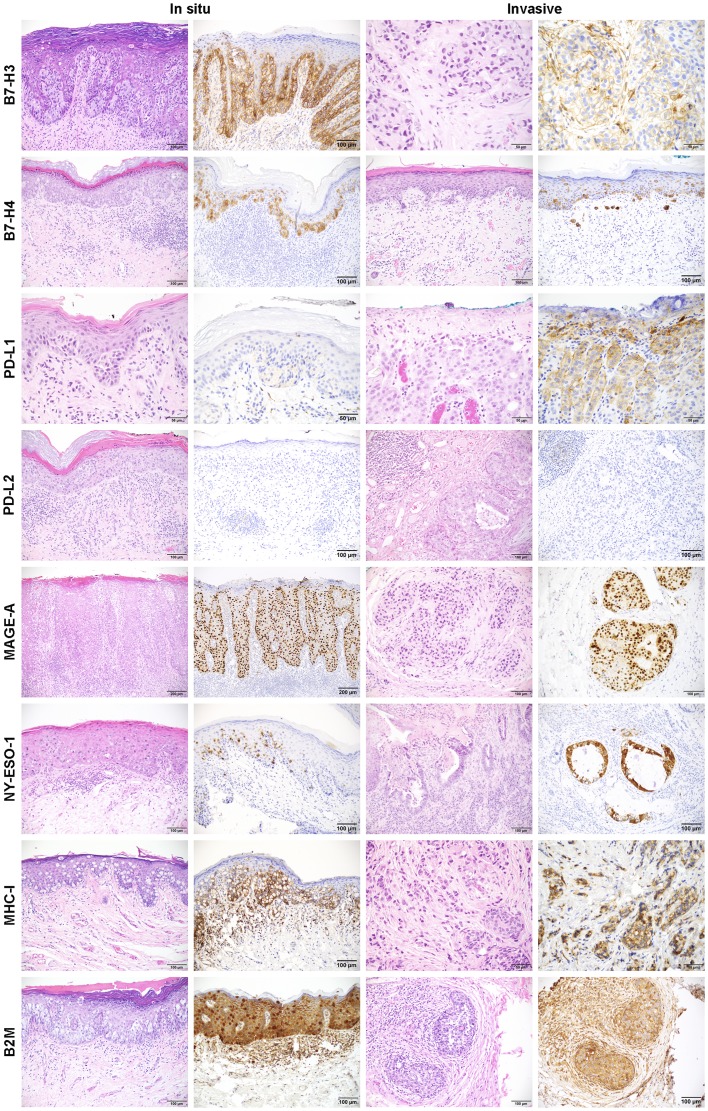
Representative images of immunohistochemistry for B7-H3, B7-H4, PD-L1, PD-L2, MAGE-A, NY-ESO-1, MHC-I, and B2M in *in situ* and invasive EMPD. IHC images for each marker along with the corresponding H&E image for each case are shown for *in situ* and invasive EMPD.

B7-H3 expression by IHC was present within the *in situ* component of 46 (93.9%) of 49 cases and in the invasive component of 17 (89.5%) of 19 cases with mean expression levels of 8.13 and 5.82, respectively (Figure 1, Supplementary Table 2). There was no significant difference in B7-H3 positive cases among the different sex, extent of disease, origin, site, treatment, and CK7/CK20 status classifications (Supplementary Table 3). B7-H4 expression was present within the *in situ* component of 40 (81.6%) of 49 cases and in the invasive component of 14 (77.8%) of 18 cases with mean expression levels of 5.90 and 7.61, respectively (Figure 1, Supplementary Table 2). Significantly more B7-H4 positive cases were seen in primary (88.6% *in situ* component, 99.3% invasive component) versus secondary disease (20.0% *in situ* component, 0.0% invasive component), in vulvar versus perianal disease (*in situ* and invasive components), and in CK7 single positive versus CK20 single positive cases (*in situ* component) (Supplementary Table 3). PD-L1 expression was present in the *in situ* component of 3 (6.1%) of 49 cases and in the invasive component of 2 (9.5%) of 21 cases with mean expression levels of 2.33 and 2.50, respectively (Figure 1, Supplementary Table 2). There was no significant difference in PD-L1 positive cases among the different sex, extent of disease, origin, site, treatment, and CK7/CK20 status classifications (Supplementary Table 3). PD-L2 expression was negative in 100% of tumor cells. B7-H3 expression in TILs was found in 43 (79.6%) of 54 cases with a median percent positive score of 1–25% (1) (Supplementary Table 1, Supplementary Table 4). The correlation between B7-H3 TIL percent positive scores and CD8 density (cells/mm^2^), measured using Kendall Tau-b rank correlation coefficient, showed a moderate positive correlation (Kendall Tau-b 0.39). PD-L1 expression in TILs was focal but found in 39 (72.2%) of 54 cases. PD-L2 expression was limited to a focal dendritic cell population and found in 4 (7.4%) of 54 cases. B7-H4 was not expressed in the TILs of any cases.

MAGE-A expression was present in the *in situ* component of 19 (38.8%) of 49 cases and in the invasive component of 10 (50.0%) of 20 cases with mean expression levels of 5.92 and 8.60, respectively (Figure 1, Supplementary Table 2). NY-ESO-1 expression was present in the *in situ* component of 8 (16.3%) of 49 cases and in the invasive component of 4 (20.0%) of 20 cases with mean expression levels of 3.81 and 4.00, respectively. There were no significant differences in MAGE-A and NY-ESO-1 positive cases among the different sex, extent of disease, origin, site, treatment, and CK7/CK20 status classifications (Supplementary Table 3).

B2M expression was present in the *in situ* component of 49 (100%) of 49 cases and in the invasive component of 16 (88.9%) of 18 cases with mean expression levels of 6.74 and 8.31, respectively (Figure 1, Supplementary Table 2). MHC-I expression was present in the *in situ* component of 38 (77.6%) of 49 cases and in the invasive component of 15 (78.9%) of 19 cases with mean expression levels of 6.38 and 6.90, respectively. There were no significant differences in B2M and MHC-I positive cases among the different sex, extent of disease, origin, site, treatment, and CK7/CK20 status classifications (Supplementary Table 3). The two metastatic EMPD tumors, both of which were from the same patient, exhibited no B2M expression and one of two also exhibited no MHC-I expression with the other expressing very low levels of MHC-I (Supplementary Figure 3).

### CK7/CK20 expression in EMPD

**Figure 2:**
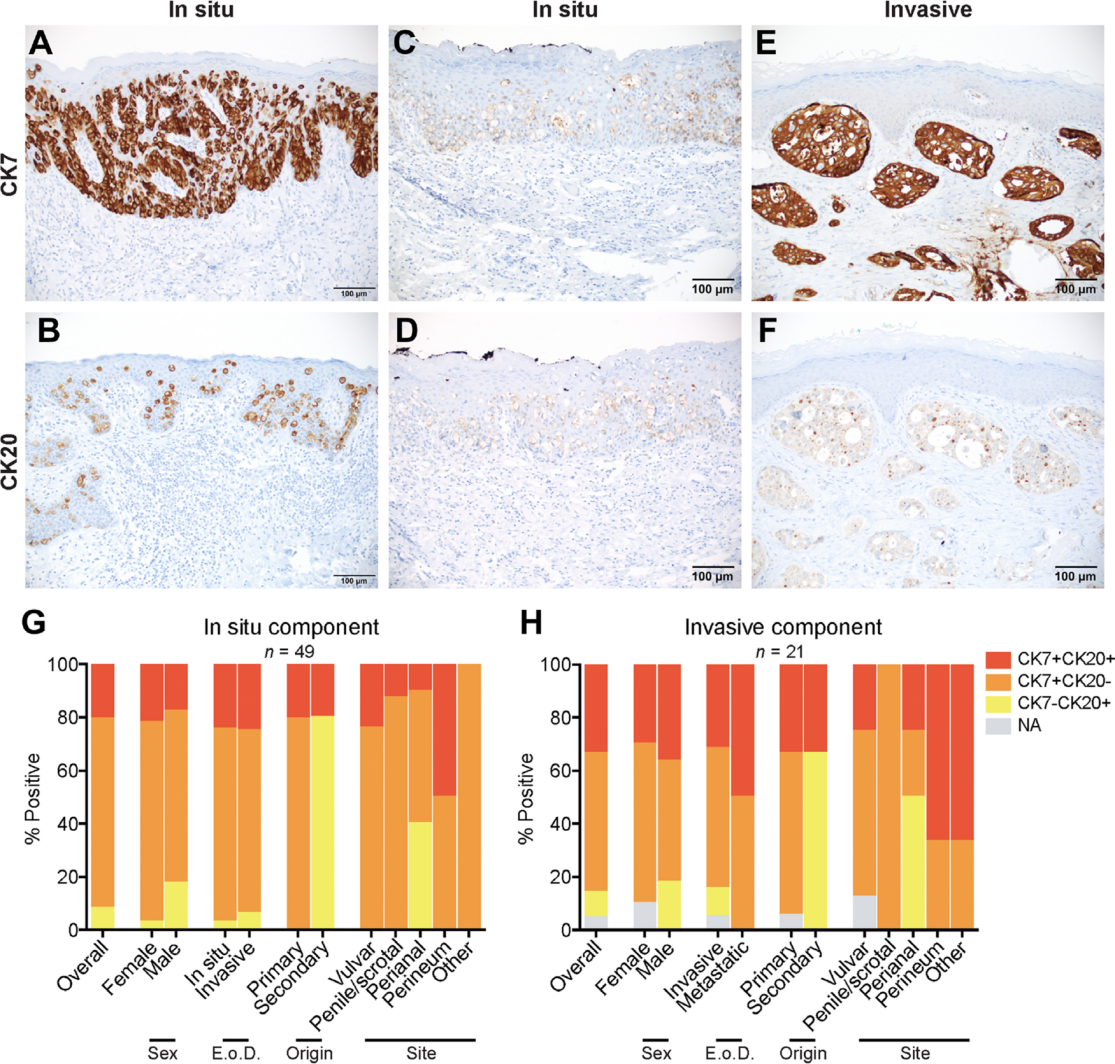
Expression and coexpression of CK7 and CK20 in EMPD. (**A**) A CK7 positive/CK20 negative *in situ* EMPD case shows strong staining intensity (3) in all tumor cells (4). (**B**) A CK20 positive/CK7 negative *in situ* EMPD case shows strong staining intensity (3) in all tumor cells (4). (**C** and **D**) A CK7 positive/CK20 positive *in situ* EMPD case shows weak staining intensity (1) in all tumor cells (4) for both markers. (**E** and **F**) A CK7 positive/CK20 positive invasive EMPD case shows a strong staining intensity (3) in all tumor cells (4) for CK7 and a weak staining intensity (1) in all tumor cells (4) for CK20. (**G**) Coexpression of CK7 and CK20 for all tumors with an *in situ* component (*n =* 49) categorized by sex, extent of disease (E o D.), origin, and site. (**H**) Coexpression of CK7 and CK20 for all tumors with an invasive component (*n =* 21) categorized by sex, extent of disease (E. o. D.), origin, and site.

CK7 expression was present in the *in situ* component of 45 (91.8%) of 49 cases and in the invasive component of 19 (90.5%) of 21 cases with mean expression levels of 11.33 and 11.53, respectively (Figure 2A, Supplementary Table 2). Significantly more CK7 positive cases were seen in primary versus secondary disease (*in situ* component) and in vulvar versus perianal disease (*in situ* component) (Supplementary Table 3). CK20 expression was present in the *in situ* component of 14 (28.6%) of 49 cases and in the invasive component of 9 (45.0%) of 20 cases with mean expression levels of 5.75 and 5.44, respectively (Figure 2B, Supplementary Table 2). 100% of secondary EMPD tumors exhibited CK20 expression, and significantly more CK20 positive cases were seen in secondary versus primary origin (*in situ* component) (Supplementary Table 3).

Several *in situ* and invasive EMPD tumors expressed both CK7 and CK20 (Figure 2C–2F). Figure 2C, 2D represents a CK7/CK20 double positive *in situ* vulvar EMPD primary tumor. Figure 2E, 2F represents a CK7/CK20 double positive invasive perineal EMPD primary tumor. Among the *in situ* component of EMPD tumors overall, 71.4% were CK7 single positive, 8.2% were CK20 single positive, and 20.4% were CK7/CK20 double positive (Figure 2G). Among the invasive component of EMPD tumors overall, 52.4% were CK7 single positive, 9.5% were CK20 single positive, and 33.3% were CK7/CK20 double positive (Figure 2H). CK7 positivity was found in a fraction of EMPD secondary to an underlying intestinal adenocarcinoma (20.0% *in situ* component, 33.3% invasive component), and CK20 positivity was found in a fraction of primary cutaneous EMPD (20.5% *in situ* component, 33.3% invasive component). All primary tumors expressed CK7 or both CK7 and CK20, and all secondary tumors expressed CK20 or both CK20 and CK7. Among the different tumor classifications, double positive CK7/CK20 expression was highest in tumors localized in the perineum (50.0% *in situ* component, 66.7% invasive component).

### Hierarchical clustering of EMPD tumors

**Figure 3:**
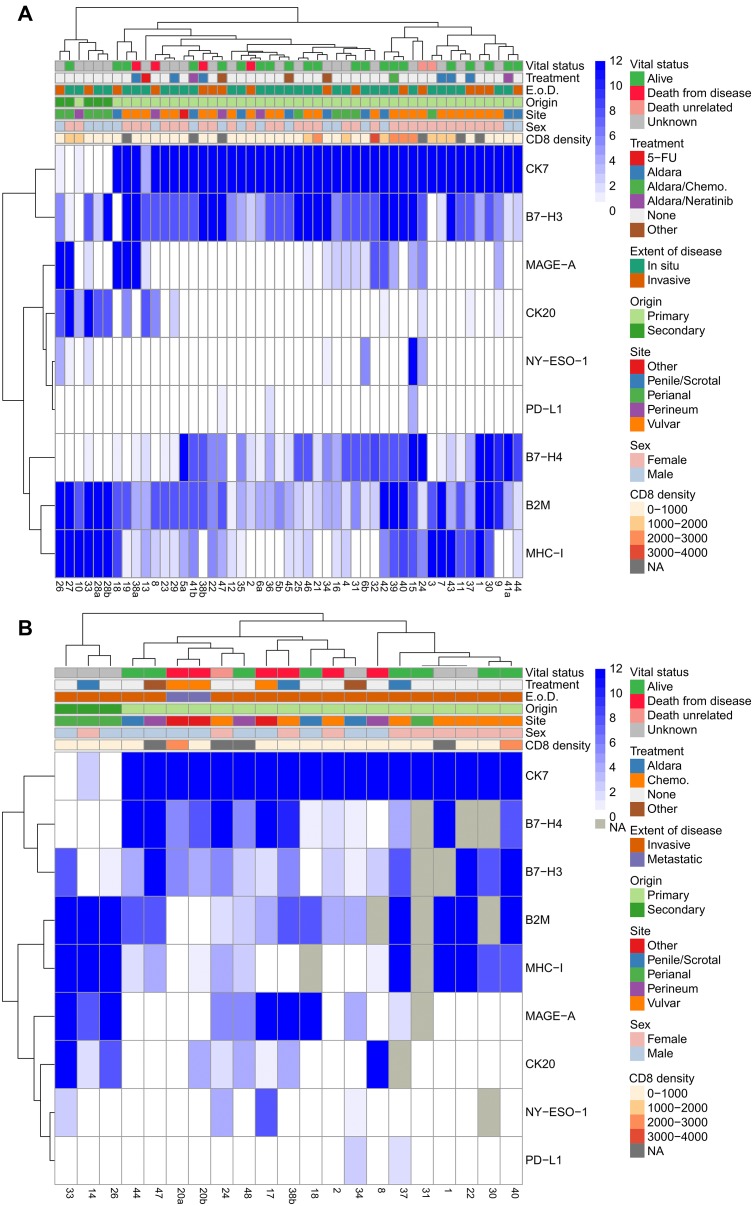
Hierarchical clustering of *in situ* and invasive EMPD. Hierarchical clustering of all immunohistochemical scores and tumor characteristics (CD8 density (cell/mm^2^), sex, site, origin, extent of disease (E. o. D.), and treatment) of all tumors with an *in situ* component (*n =* 49) (**A**) and all tumors with an invasive component (*n =* 21) (**B**). The patient numbers are listed below the heatmap. Any patient number followed by a/b distinguishes between multiple resections from the same patient. Missing values are labeled as NA and not utilized in the clustering.

**Figure 4:**
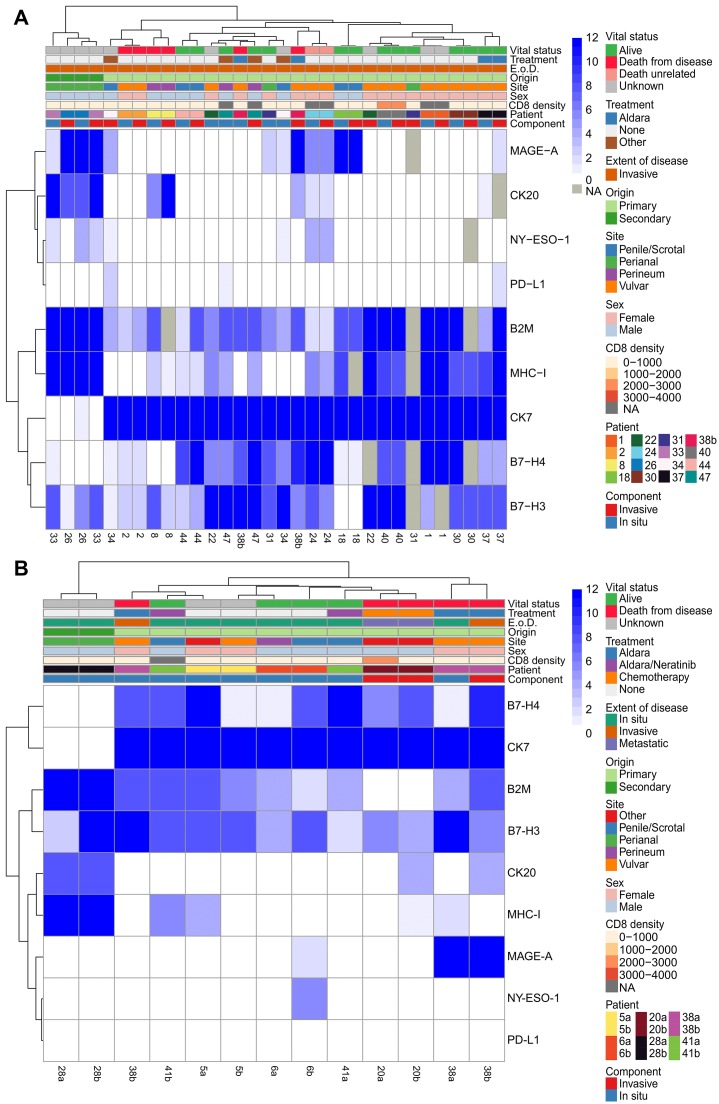
Hierarchical clustering of *in situ* and invasive components of the same tumor and multiple longitudinal resections. Hierarchical clustering of all immunohistochemical scores and tumor characteristics (CD8 density (cell/mm^2^), sex, site, origin, extent of disease (E. o. D.), treatment, and component (comp.)) of all tumors with both an *in situ* and invasive component (*n =* 16) (**A**) and all patients with two resections (*n =* 6) (**B**). Case a represents an older resection while cases b represents the more recent resection for patients with two resections. Missing values are labeled as NA and not utilized in the clustering.

Hierarchical clustering was conducted between the *in situ* component of all EMPD tumors (*n =* 49) (Figure 3A), the invasive component of all EMPD tumors (*n =* 21) (Figure 3B), EMPD tumors with both *in situ* and invasive components (*n =* 16) (Figure 4A), and patients with two resections (*n =* 6) (Figure 4B). Additional heatmaps ordered by vital status and extent of disease for the *in situ* component (Supplementary Figure 4A, 4B) and the invasive component (Supplementary Figure 4C, 4D) of all EMPD tumors were generated to further compare the expression of the studied biomarkers within tumors with similar clinical characteristics. The patient number followed by “a” and “b” was used to distinguish between two resections from the same patient with “a” representing the earliest resection and “b” representing the more recent resection.

Within the *in situ* component of EMPD tumors, tumors of secondary origin localized in the perianal region showed higher scores for B2M, MHC-I, and CK20 and lower B7-H4 expression (Figure 3A) compared with primary EMPD. CK7 expression was high in all primary EMPD tumors with the exception of one primary tumor treated with 5-FU and one of four primary tumors localized in the perineum. The correlation between CD8 density (counts/mm^2^) and expression of each marker in the *in situ* component of EMPD tumors, measured using Kendall Tau-b rank correlation coefficient, showed a moderate positive correlation between CD8 and MAGE-A and between CD8 and MHC-I (Supplementary Figure 5). A weaker positive correlation with CD8 was found for both NY-ESO-1 and B2M. The correlation between TIL B7-H3 and expression of each marker in the *in situ* component of EMPD tumors, measured using Kendall tau-b, showed only a weak positive correlation with B2M expression in tumor (Supplementary Figure 5). There was no correlation between vital status and any marker expression in tumor as measured by Kendall Tau-b. There was weak-to-moderate positive correlation between vital status & TIL B7-H3 (Kendal tau-b 0.46). There was no correlation between vital status and either PD-L1 or PD-L2 in TILs (Kendall Tau-b and Fisher exact test).

Among the invasive component of EMPD tumors, higher B2M, MHC-I, MAGE-A, and CK20 and no B7-H4 and PD-L1 expression was observed in tumors of secondary origin localized in the perianal region (Figure 3B) compared to primary cutaneous EMPD. The patient with two metastatic tumor biopsies expressed low to no MHC-I, were negative for B2M, PD-L1, NY-ESO-1, and MAGE-A, and were positive for B7-H3 and B7-H4. The 5 patients who died from EMPD all had low to no MHC-I expression in their invasive tumor components. The NA values were a result of no remaining tumor in serial sections of the block.

The correlation between marker expression within the *in situ* and invasive tumor components of invasive EMPD cases was explored using hierarchical clustering (Figure 4A). Although some cases clustered together (1, 2, 8, 18, 24, 26, 30, 37, 40, 44), the expression levels of some markers differed between the *in situ* and invasive components of a single case. For example, EMPD tumor 47 had high expression of B7-H4 (12) in its invasive component but medium expression of B7-H4 (6) in its *in situ* component (Figure 1, B7-H4 invasive case). EMPD tumor 38b had high expression of MAGE-A (12) in its invasive component but was negative for MAGE-A (0) in its *in situ* component. This tumor was also CK20 positive in its invasive component, but CK20 negative in its *in situ* component. In contrast, tumor 38b had higher B7-H3 expression (12) in its *in situ* component than its invasive component (4). Correlation between marker expression in the *in situ* and invasive tumor component by case, measured using Spearman rank coefficient, showed high positive correlation in the majority of cases when considering all markers together with the exception of case 34 and 38b (Supplementary Figure 6). Similarity between the *in situ* and invasive tumor component of each case by marker, measured using cosine similarity, showed the highest similarity between the two components for CK7, followed by MHC-I, B7-H4, B2M, CK20, and B7-H3 in order of least to greatest change (Supplementary Figure 7). Comparatively, MAGE-A and NY-ESO-1 showed the lowest similarity.

The correlation in marker expression between two resections from the same patient over time using hierarchical clustering revealed that while some tumors continue to have similar expression levels over time, others have differences (Figure 4B). Only one of six patients, patient 28, retained positivity for the same set of markers over time. Patient 6 gained MAGE-A and NY-ESO-1 tumor cell expression, and patient 20 gained CK20 expression. Patients 20 and 41 gained tumor cell expression of MHC-I, and patients 5 and 38 lost tumor cell expression of MHC-I. Overall, despite variations in the expression levels of B7-H3, B7-H4, B2M, and CK7 among the two resections from the same patient, the positivity of these markers was conserved in the tumor cells of all patients over time. The correlation in marker expression between two resections from the same patient, measured using Spearman rank coefficient, showed moderate to high correlation between resections when considering all 10 markers simultaneously with patient 28 showing the highest correlation and patient 38 showing the lowest correlation (Supplementary Figure 8). Similarity between multiple resections from the same patient by marker, measured using cosine similarity, showed at least a moderate level of similarity for all markers with CK7 showing the least change between the two resections followed by B2M, CK20, B7-H3, MHC-I, and B7-H4 in order of least to greatest change (Supplementary Figure 9).

### Coexpression of B7 family members and cancer/testis antigens within a single case

**Figure 5:**
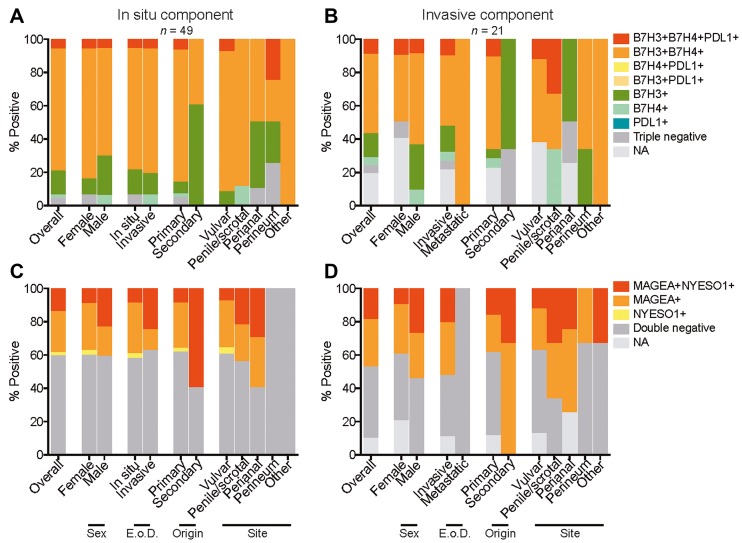
Coexpression of B7 family members and cancer/testis antigens in EMPD. Coexpression of B7 family members B7-H3, B7-H4, and PD-L1 categorized by sex, extent of disease (E o D.), origin, and site for all tumors with an *in situ* component (**A**) and all tumors with an invasive component (**B**). Coexpression of cancer/testis antigens MAGE-A and NY-ESO-1 categorized by sex, extent of disease (E. o. D.), origin, and site for all tumors with an *in situ* component (**C**) and all tumors with an invasive component (**D**). NA refers to cases in which there were no remaining tumor cells in serial sections to be scored.

Several EMPD cases co-expressed a combination of B7-H3, B7-H4, and PD-L1. Among the *in situ* tumor component, 3 (6.1%) of 49 were B7-H3/B7-H4/PD-L1 triple positive, 36 (73.5%) of 49 were B7-H3/B7-H4 double positive, 7 (14.3%) of 49 were B7-H3 single positive, and 1 (2.0%) of 49 were B7-H4 single positive overall (Figure 5A). Among the invasive tumor component, 2 (9.5%) of 21 were B7-H3/B7-H4/PD-L1 triple positive, 10 (47.6%) of 21 were B7-H3/B7-H4 double positive, 3 (14.3%) of 21 were B7-H3 single positive, and 1 (4.8%) of 21 were B7-H4 single positive (Figure 5B). B7-H3 single positivity was observed in a larger number of cases in comparison to B7-H4 single positivity.

Coexpression of MAGE-A and NY-ESO-1 was also observed in the EMPD cases. Among the *in situ* tumor component, 7 (14.3%) of 49 were MAGE-A/NY-ESO-1 double positive, 12 (24.5%) of 49 were MAGE-A single positive, and 1 (2.0%) of 49 was NY-ESO-1 single positive (Figure 5C). Among the invasive tumor component, 4 (19.0%) of 21 tumors were MAGE-A/NY-ESO-1 double positive and 6 (28.6%) of 21 were MAGE-A single positive (Figure 5D). The two metastatic tumors were negative for both MAGE-A and NY-ESO-1, and MAGE-A single positivity was observed in a larger number of cases in comparison to NY-ESO-1 single positivity.

## DISCUSSION

We report for the first time the differential expression of B7 family members in tumor cells of EMPD in a cohort of predominantly primary cutaneous disease. Our results show that the majority of cutaneous EMPD cases have tumor expression of B7-H3 and B7-H4 with few cases expressing PD-L1 and no cases showing tumor expression of PD-L2. These findings are true for both *in situ* and invasive disease. B7 family member expression does not appear correlative with treatment or sex. For the most part, B7-H3 and B7-H4 expression is concordant between the *in situ* and invasive components of an individual patient with many cases showing co-expression of these markers. Two metastatic lesions from the same patient showed that B7-H3 and B7-H4 were expressed in both metastatic deposits (the primary tumor was not available for comparison).

B7-H4 is more frequently expressed in primary cutaneous disease compared to EMPD secondary to cutaneous involvement by a colorectal primary tumor. Based on this finding, strong B7-H4 expression may suggest primary cutaneous origin in cases with equivocal or unknown site of origin; however, a larger series of secondary EMPD cases would be helpful to validate this observation. We also report that cutaneous expression of B7-H4 in the normal skin is limited to the follicular root sheath, sebaceous lobule epithelium, and an epithelial component of the eccrine duct (Supplementary Figure 1). While expression of B7-H4 is limited to these anatomic structures in the skin, the origin of EMPD still remains uncertain.

Expression of B7-H4 is generally regarded as protumorigenic often correlating with decreased T cell infiltrates with aberrant macrophage function [28–31], higher stage of disease [32], increased lymph node involvement [33], and lower survival [34–41]. Although some reports show contrasting results and discrepant staining patterns in TIL subsets [42–44], it is possible that cellular context and other features of the tumor microenvironment impact outcome. While expression within the leukocytes is best assessed with multiplexed staining modalities with quantitative digital analysis [45], we did not see B7-H4 expression within associated leukocytes or within stromal macrophage components (data not shown) with chromogenic detection on whole tissue sections. In fact, B7-H4 expression was restricted to the tumor and anatomic structures of the skin described above. These data are consistent with reports that B7-H4 protein expression is tightly regulated with leukocyte protein expression often requiring significant stimulation [42, 43].

We have shown that B7-H3 is widely expressed across our EMPD cohort of both primary and secondary disease. B7-H3 expression is relatively broad among normal tissues with increased levels in a diverse group of cancers including lung, kidney, breast, prostate, and brain [46, 47]. The majority of studies show that B7-H3 functions as a co-inhibitory molecule with engagement resulting in inhibition of T cell proliferation and decreased secretion of cytokines such as TNF-alpha and IFN-gamma [46, 48–51]. B7-H3 can be expressed by numerous cell lineages; however, the function of B7-H3 in antigen presenting cells (APCs) is to inhibit T-cell activation via IL-2 suppression [51]. B7-H3 has many other proposed functions in tumor biology including contributions to tumor growth [52], metastasis [53] and drug resistance [54]. In our series, we show broad and diffuse B7-H3 expression across the cohort with relatively diffuse expression in the tumor associated leukocytes (Supplementary Table 1, Supplementary Table 4). We also showed a weak positive correlation between TIL B7-H3 and B2M expression in tumor, a weak-to-moderate positive correlation between TIL B7-H3 and vital status, and a moderate positive correlation between TIL B7-H3 and CD8 density.

Within the cohort, EMPD tumor cells rarely express PD-L1, and PD-L2 was negative in all tumor cells. We found occasional PD-L2 expressed by leukocytes, morphologically within the APC lineage. PD-L1 was also focally seen in associated TILs (Supplementary Table 4); however, the levels were not appreciable and better quantified with other technologies allowing for subset assessment and more accurate quantification [45]. There was no correlation between TIL PD-L1 or PD-L2 and vital status. Our PD-L1 findings confirm two recent studies performed by Karpathiou et al. and Mauzo et al. that also report low levels of PD-L1 in EMPD tumor cells [55, 56].

We also identified expression of CTAs MAGE-A and NY-ESO-1 on tumor cells of EMPD of both primary and secondary origin and in *in situ* and invasive disease but not in the lone patient with metastatic disease. MAGE-A expression showed the highest positive correlation with CD8 density among all studied markers in tumor. MAGE-A and NY-ESO-1 are generally regarded as good candidates for immunotherapy as they show limited expression in normal tissues with aberrant expression in a broad array of tumor tissues [23, 57, 58]. Relevant to this work, it is well known that MAGE-A1 and NY-ESO-1 are expressed in colorectal carcinoma [59], but this is the first report of primary EMPD expressing CTAs. Importantly, CTAs are generally regarded as immunogenic proteins, and both MAGE-A and NY-ESO-1 are currently targets for cancer immunotherapy. It is well known that the anti-Mage-A monoclonal 6C1 utilized in this study shows immunoreactivity for numerous homologous Mage family members including MAGE-A1, MAGE-A2, MAGE-A3, MAGE-A4, MAGE-A6, MAGE-A10, and MAGE-A12, and further subclassification would require isoform specific rt-PCR or similar studies [60].

The common expression of B2M and MHC-I on tumor cells of EMPD reported herein demonstrates that EMPD retains the cellular antigen presenting machinery that is widely regarded as beneficial for immune targeting suggesting that EMPD patients have a greater likelihood of responding to immunotherapy. We also report a patient with metastatic EMPD to lymph node showing no expression of B2M or MHC-I in one lesion and no expression of B2M and focal weak expression of MHC-I in another metastasis. While the primary tumor was not available for comparison, loss of B2M and MHC-I have been reported as mechanisms of immune resistance [61, 62].

This study confirms prior reports showing that cutaneous EMPD shows strong CK7 immunoreactivity and disease secondary to colorectal carcinoma shows strong CK20 staining [63–67]. One patient with vulvar EMPD showed weak CK7 staining and strong CK20 reactivity. Interestingly, this was the only patient to have received 5-FU therapy prior to biopsy. Of the six patients with EMPD secondary to a colorectal primary, all showed strong CK20 staining and two had concurrent, weak CK7 staining.

The majority of EMPD patients have non-invasive disease restricted to the epidermis and adnexal epithelium with a small percentage showing invasion. With localized disease, accessibility of locoregional therapies delivered by topical administration or injection may be a desirable or more effective alternative to surgical management. We report that EMPD expresses high levels of B7 family members B7-H3 and B7-H4 with lower levels of CTAs MAGE-A and NY-ESO-1, very focal expression of PD-L1, and no expression of PD-L2 in addition to high levels of antigen presenting machinery molecules B2M and MHC-I in this cohort. A search of clinicaltrials. gov for interventional clinical trials identified forty-two B7-H3, one B7-H4, forty-six MAGE-A, and one hundred twenty-four NY-ESO-1 trials that are either not yet recruiting, recruiting, enrolling by invitation, active, suspended/terminated, or completed. Of these clinical trials, there are fifteen B7-H3, one B7-H4, ten MAGE-A, and thirty-seven NY-ESO-1 trials that are currently recruiting patients for various conditions (none for EMPD). Given our findings, inclusion of EMPD patients in these clinical trials or the development of new clinical trials such as B7-H3 or B7-H4 targeted therapy in combination with MAGE-A or NY-ESO-1 targeted therapy may prove to be successful in the management of EMPD.

## MATERIALS AND METHODS

### Case selection

Forty-eight patients with *in situ* or invasive EMPD were identified, and 57 surgically resected, formalin-fixed, paraffin-embedded (FFPE) EMPD specimens were obtained from the archives of Memorial Sloan Kettering Cancer Center (MSKCC). This study was approved by the MSKCC institutional biospecimen review board. Four-micron thick tissue sections were stained with hematoxylin and eosin (H&E) and reviewed by a dermatopathologist (T. Hollmann) to confirm the diagnosis of *in situ*, invasive, or metastatic EMPD and to select a representative tumor block in cases where more than one tissue block was available per excision. Two tumor blocks from three cases were selected and later averaged, resulting in a final count of 54 unique EMPD specimens labeled by sample ID 1-48. Six patients had two resections. The patient files were reviewed, and patient characteristics including age, sex, extent of disease, origin and site of disease, prior medicinal treatments, and vital status are summarized in Table 1. Extent of disease was characterized as *in situ*, invasive, or metastatic. Primary versus secondary disease was determined by searching patient histories for colorectal cancer. The four other sites consist of metastatic disease in the lymph nodes, cutaneous metastatic disease on the abdomen, *in situ* disease on the buttock, and invasive disease on the thigh. The other treatments consist of an unknown cream used by the patient, Lenalidomide for multiple myeloma, and FOLFOX-Avastin for metastatic colon cancer. A vital status of unknown was recorded for patients with a last follow-up appointment of greater than two years.

### Immunohistochemical staining

Immunohistochemical staining for B2M, B7-H3, B7-H4, CD8, CK7, CK20, MAGE-A, MHC-I, NY-ESO-1, PD-L1, and PD-L2 (Table 2) was performed on 4-micron thick FFPE serial sections using an automated staining system (Leica Bond). Antigen retrieval was conducted for 30 minutes using Bond epitope retrieval solution 2 (EDTA, pH 9.0) followed by incubation of the primary antibody for 30 minutes for all markers. Appropriate positive controls were included in each staining run (B7-H3, placenta (cytotrophoblast); B7-H4, normal hair follicle; PD-L1, placenta; PD-L2, normal tonsil; MAGE-A, placenta; NY-ESO-1, normal testicle; B2M, normal skin; MHC-I, normal skin; CK7, normal breast; CK20, normal colon; CD8, normal tonsil) (Supplementary Figure 1). The B7-H3 and B7-H4 clones were independently validated for specificity [68].

**Table 2:** Antibodies used for immunohistochemistry

Primary antibody	Clone	Vendor	Catalog number	Concentration (μg/mL)
B2M	Polyclonal	Dako	A007202	1
B7-H3	D9M2L	Cell Signaling Technology	14058S	0.125
B7-H4	D1M8I	Cell Signaling Technology	14572S	5.2
CD8	C8/144B	Dako	M7103	1.5
CK7	OV-TL-12/30	Dako	M7018	0.245
CK20	Ks20.8	Dako	M7019	0.0425
MAGE-A	6C1	Thermo Fisher Scientific	35-6300	10
MHC-I	A4	eBioscience	14-9958	2.5
NY-ESO-1	E978	Thermo Fisher Scientific	35-6200	1
PD-L1	E1L3N	Cell Signaling Technology	13684S	2
PD-L2	D7U8C	Cell Signaling Technology	82723S	5

Abbreviations: B2M, beta-2-microglobulin; CK, cytokeratin; PD-L1, programmed death-ligand 1; PD-L2, programmed death-ligand 2; MAGE-A, melanoma-associated antigen A; MHC-I, major histocompatibility complex class I; NY-ESO-1, New York esophageal squamous cell carcinoma 1.

### Assessment of immunohistochemical staining

The IHC stains were evaluated to determine the expression of each marker in tumor cells using a semi-quantitative scoring method based on the percentage of positive tumor cells and the staining intensity of each marker in tumor cells. Positivity for B7-H3, B7-H4, PD-L1, PD-L2, B2M, and MHC-I was defined by membranous expression. Positivity for NY-ESO-1 and MAGE-A was defined by membranous and/or cytoplasmic expression. Positivity for CK7 and CK20 was defined by any staining in the tumor cells. Slides were scored blindly by T. Hollmann and M. Pourmaleki. The percentage of positive tumor cells was estimated and categorized as negative (0), 1-25% (1), 26-50% (2), 51-75% (3), or 76-100% (4). IHC staining intensity was scored as negative (0), weak (1), moderate (2), or strong (3). Examples of 0-3 staining intensity for B7-H4 are included in Supplementary Figure 2. The product of percentage of positive tumor cells and IHC staining intensity, ranging from 0 to 12, was calculated and utilized as the final reported score for each marker (Supplementary Table 1). For the three cases that had two tumor blocks stained (5b, 9, 38b), scores for each marker were averaged. Some figures and tables have slightly differing specimen numbers due to lack of remaining tumor cells in the serial sections of some cases. The expression of B7-H4, PD-L1, and PD-L2 in tumor infiltrating lymphocytes (TILs) in each case was assessed and reported as positive or negative. The percentage of B7-H3 positive TILs in each case was estimated and categorized as negative (0), 1-25% (1), 26-50% (2), 51-75% (3), or 76-100% (4). The density of CD8 positive cells within 500 microns of the epidermis in *in situ* cases or throughout the sample in invasive cases was quantified using Halo Image Analysis Software (Indica Labs) and reported as cells per millimeter squared (Supplementary Table 1).

### Hierarchical clustering and statistical analysis

Clustering of both markers and cases was done using standard hierarchical clustering (R function hclust) with complete linkage, and for the distance function the Manhattan metric was used. Missing values were labeled as NA in R and not utilized in the clustering. Correlation between marker expression in the *in situ* and invasive tumor component by case and between two resections from the same patient was measured using Spearman rank coefficient. Similarity in marker expression between the *in situ* and invasive tumor component of each case and between two resections from the same patient by marker was measured using cosine similarity. To assess for statistically significant differences in marker percent positive among the various tumor characteristics, p-values were computed according to a permutation test, in which labels between the groups being compared in each category were randomly shuffled for 1000 iterations. A minimum of three cases in each group is being compared, and a total of 10 cases across groups were required for each permutation test. P-values across markers for any two categories being compared were corrected for multiple hypothesis testing according to the Benjamini-Hochberg procedure at 5% false discovery rate (FDR). Correlation between CD8 density and expression of each marker in tumor and TIL B7-H3 was measured using Kendall Tau-b rank correlation coefficient. Correlation between TIL B7-H3 and expression of each marker in tumor was measured using Kendall Tau-b rank correlation coefficient. Lastly, vital status was categorized into two binary categories (0 = dead, 1 = alive) by combining “Death from disease” & “Death unrelated to disease” and removing any status of “Unknown” to measure the correlation between vital status and expression of each marker in tumor and TIL B7-H3/PD-L1/PD-L2 using Kendall Tau-b rank correlation coefficient.

## SUPPLEMENTARY MATERIALS










