# Supplementary material for: Extramammary Paget disease shows differential expression of B7 family members B7-H3, B7-H4, PD-L1, PD-L2 and cancer/testis antigens NY-ESO-1 and MAGE-A
Source: Oncotarget. 2019 Oct 22;10(58):6152–67. doi: 10.18632/oncotarget.27247 (PMC6817453; doi:10.18632/oncotarget.27247)
Supplement: Supplementary file 1 [file oncotarget-10-6152-s001.pdf]

Extramammary Paget disease shows differential expression of B7 family members B7-H3, B7-H4, PD-L1, PD-L2 and cancer/testis antigens NY-ESO-1 and MAGE-A

SUPPLEMENTARY MATERIALS

Supplementary Table 1: Patient characteristics and marker scores by sample. See Supplementary Table 1

Supplementary Table 2: Immunohistochemical staining results. See Supplementary Table 2

Supplementary Table 3: Statistically significant differences in marker percent positive between clinical categories. See Supplementary Table 3

Supplementary Table 4: B7-H3, PD-L1, and PD-L2 positivity in TILs. See Supplementary Table 4

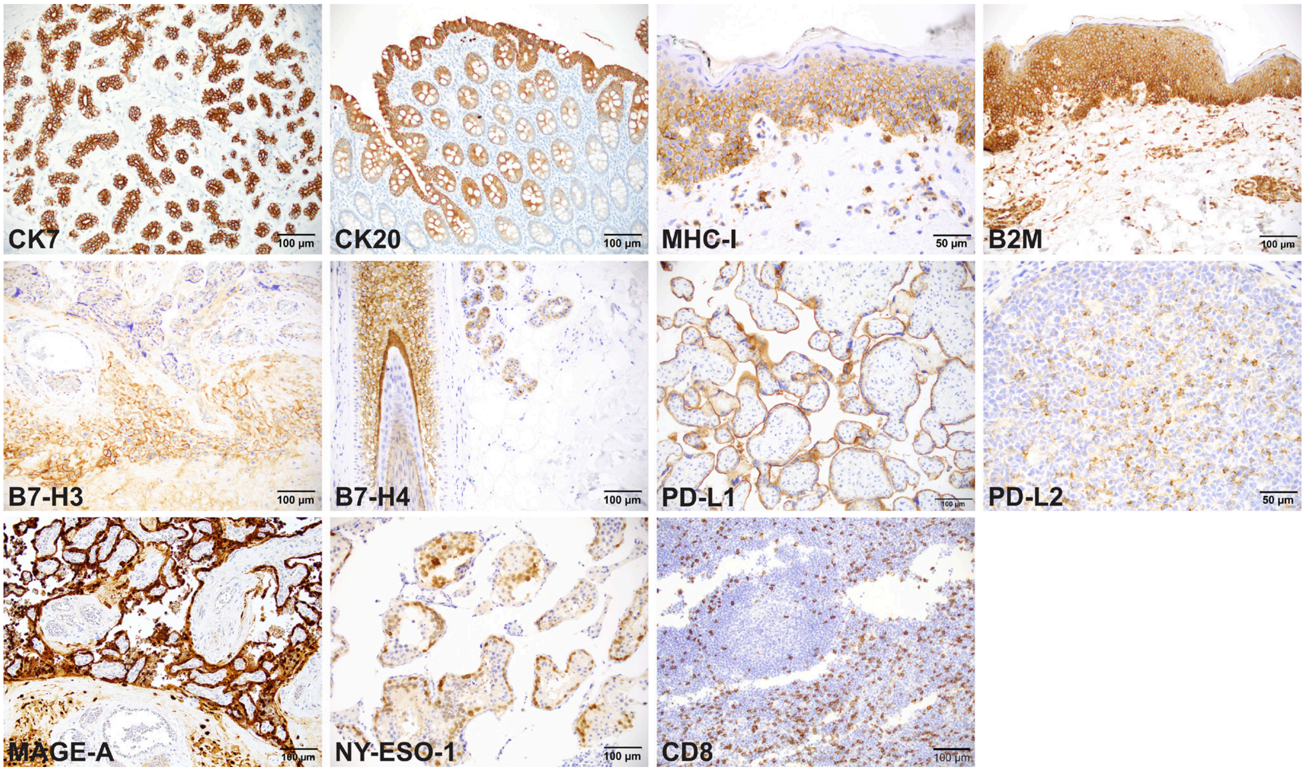

Supplementary Figure 1: Representative images of immunohistochemistry for CK7, CK20, MHC-I, B2M, B7-H3, B7-H4, PD-L1, PD-L2, MAGE-A, NY-ESO-1, and CD8 in controls. Controls include: CK7, normal breast; CK20, normal colon; MHC-I, normal skin; B2M, normal skin; B7-H3, placenta (cytotrophoblast); B7-H4, normal hair follicle; PD-L1, placenta; PD-L2, normal tonsil; MAGE-A, placenta; NY-ESO-1 normal testicle; CD8, normal tonsil.

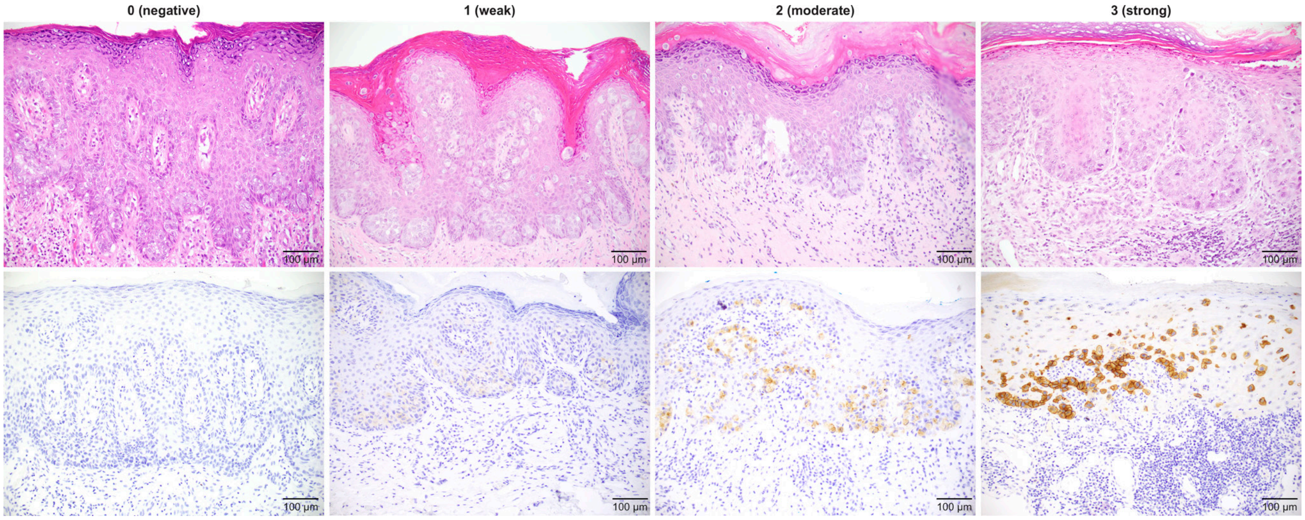

Supplementary Figure 2: Example of IHC staining intensity scoring system. Example images of IHC staining intensity for B7-H4 with corresponding H&E for a 0 (negative), 1 (weak), 2 (moderate), and 3 (strong) sample.

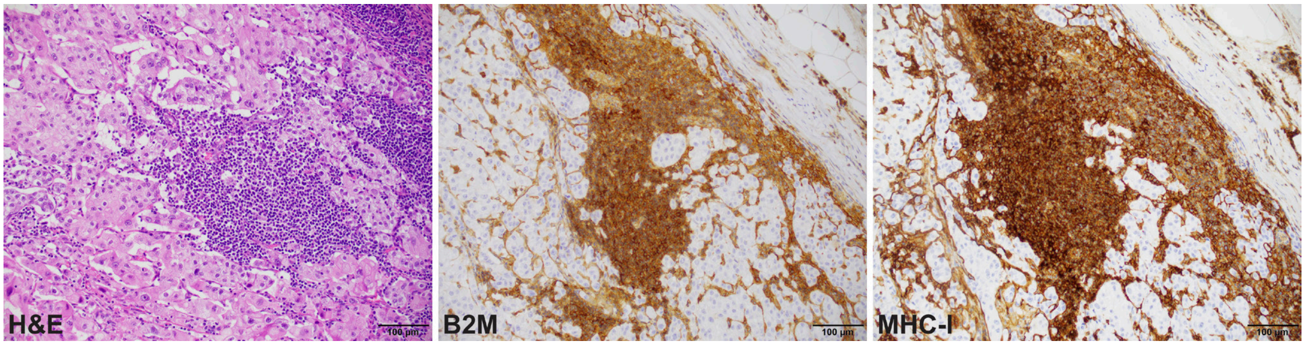

Supplementary Figure 3: Loss of B2M and MHC-I in patient with metastatic EMPD in the lymph node.

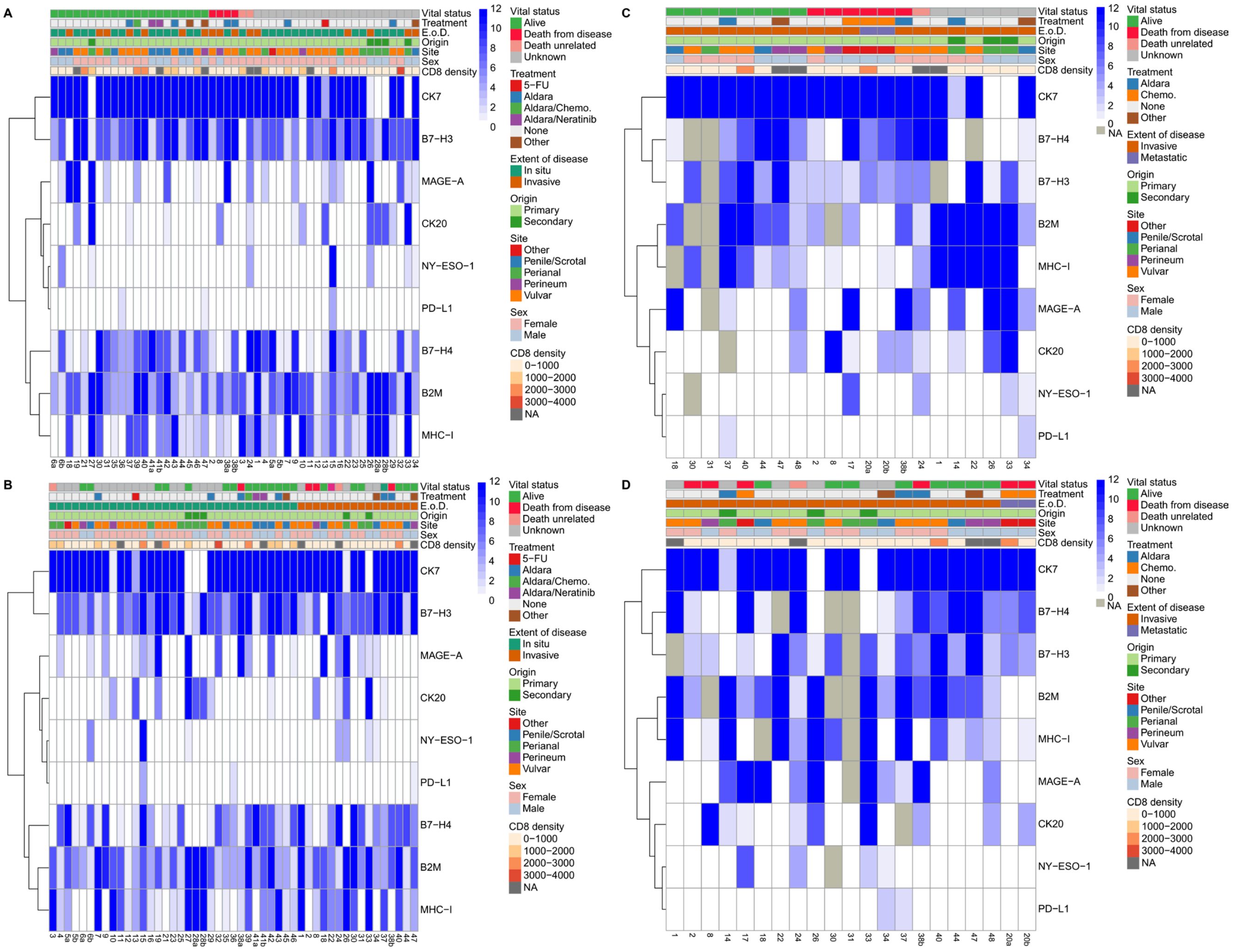

Supplementary Figure 4: Hierarchical clustering of markers in the in situ and invasive components of EMPD tumors. The expression of the markers within tumors with similar characteristics was explored by ordering the patient samples by vital status (**A**) and extent of disease (E.o.D.) (**B**) for the *in situ* component of all EMPD tumors and also by vital status (**C**) and E.o.D. (**D**) for the invasive component of all EMPD tumors.

Marker	Kendall Tau-b	
	CD8 density	TIL B7-H3
CK7	0.032	-0.2133
CK20	0.014	0.194
B7-H3	0.21	0.2169
B7-H4	0.24	-0.02115
PD-L1	0.019	-0.1161
MAGE-A	0.54	-0.1307
NY-ESO-1	0.33	-0.07539
B2M	0.31	0.3436
MHC-I	0.45	0.1376

Supplementary Figure 5: Correlation between CD8 density and TIL B7-H3 with marker expression in tumor. CD8 density values are counts of CD8 positive cells per mm2 within 500 pm of the epidermis. Correlation is measured according to the Kendall Tau-b rank correlation coefficient.

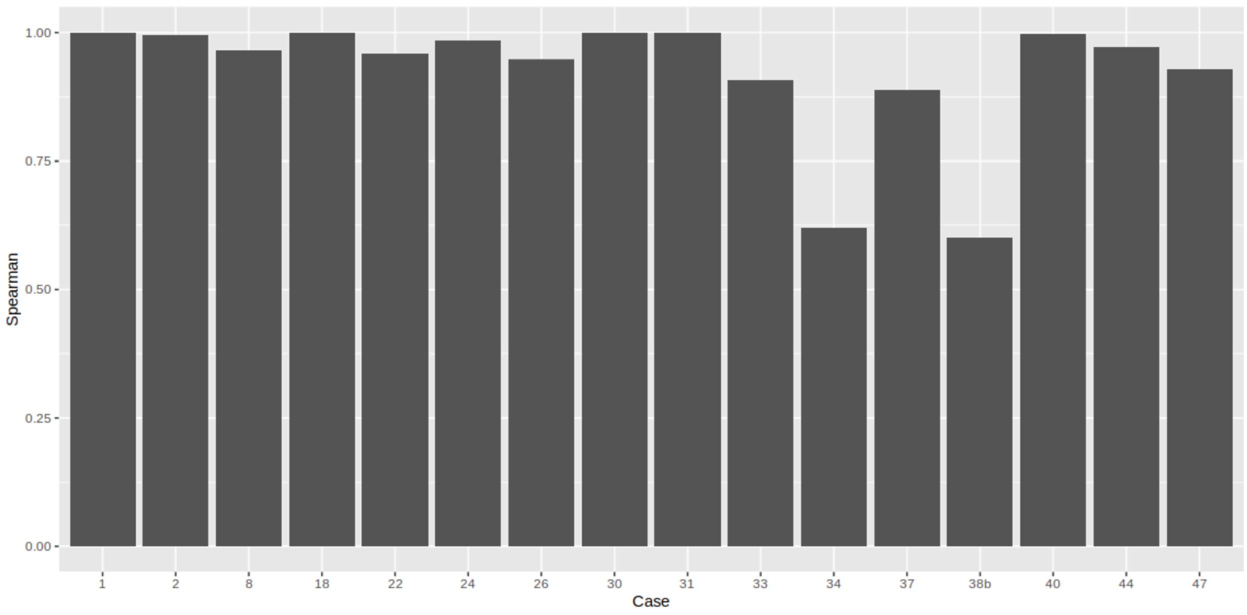

**Patient**	1	2	8	18	22	24	26	30
**Spearman**	1.00	0.996	0.965	1.00	0.958	0.984	0.948	1.00

**Patient**	31	33	34	37	38b	40	44	47
**Spearman**	1.00	0.908	0.619	0.888	0.601	0.996	0.972	0.930

Supplementary Figure 6: Correlation of marker expression between the *in situ* and invasive components by patient. For each patient (n = 16),the correlation between the *in situ* and invasive component across all markers measured by the Spearman rank coefficient is shown. Most patients show high positive correlation between the in situ and invasive component across all markers. Patients 34 and 38b show relatively weak correlation in comparison.

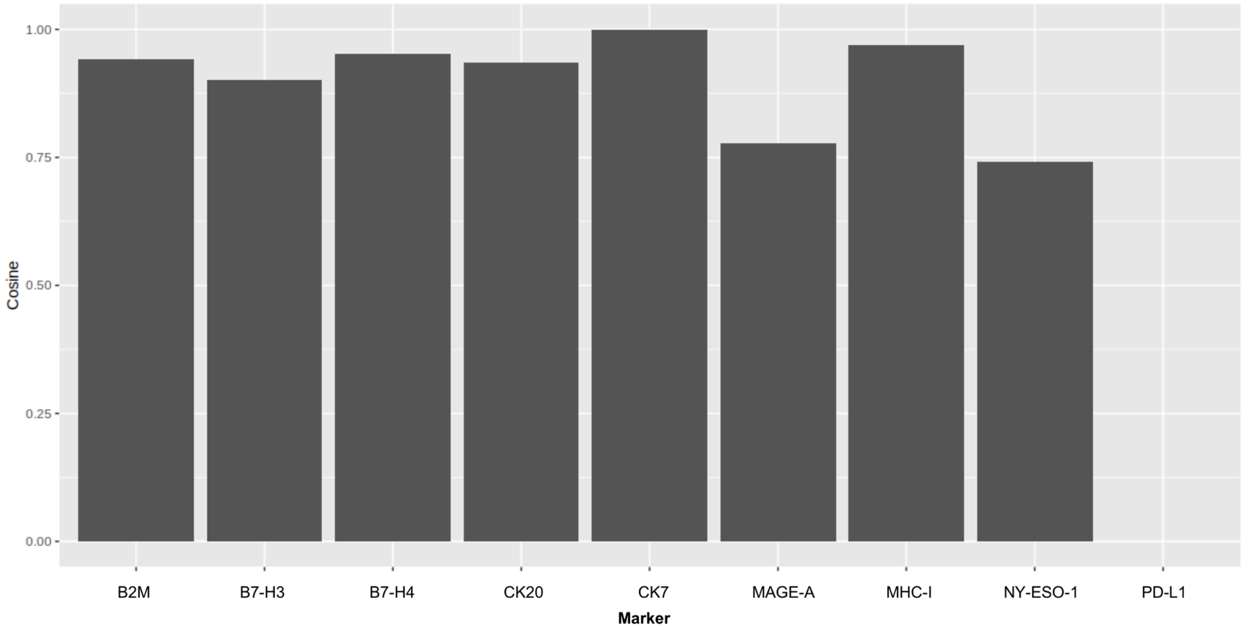

**Marker**	CK7	CK20	B7-H3	B7-H4	PD-L1	MAGE-A	NY-ESO-1	B2M	MHC-I
**Cosine**	0.9998	0.9350	0.9018	0.9518	0	0.7772	0.7415	0.9426	0.9699

Supplementary Figure 7: Similarity between the in situ and invasive component by marker. For each marker, the variation between the in situ and invasive components across all cases was measured according to cosine similarity. CK7 and MHC-I showed the highest similarity between the *in situ* and invasive component, while MAGE-A and NY-ESO-1 had the lowest similarity. IHC scores for PD-L1 were zero for most cases.

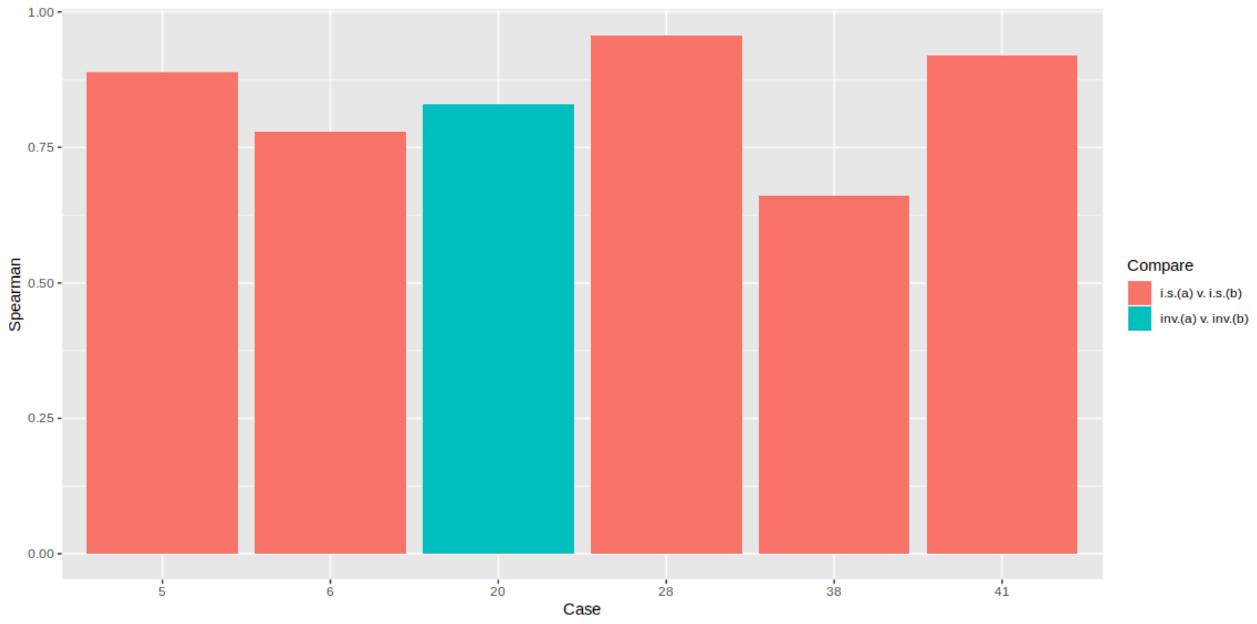

**Patient**	5	6	20	28	38	41
**Spearman**	0.889	0.779	0.830	0.957	0.662	0.919

Supplementary Figure 8: Correlation of marker expression between two resections from the same patient. For patients who had two resections ($n = 6$), the correlation between the older resection (a) and the more recent resection (b) as measured by Spearman rank coefficient is shown. In the legend, "i.s.(a) v. i.s.(b)" denotes the comparison between the *in situ* component from resection a versus the in situ component from resection b. Similarly, "inv.(a) v. inv.(b)" denotes the comparison between the invasive component from resection a versus the invasive component from resection b. Most patients show moderate to high correlation between resections when considering all markers together. Patient 38 shows the lowest correlation between resections.

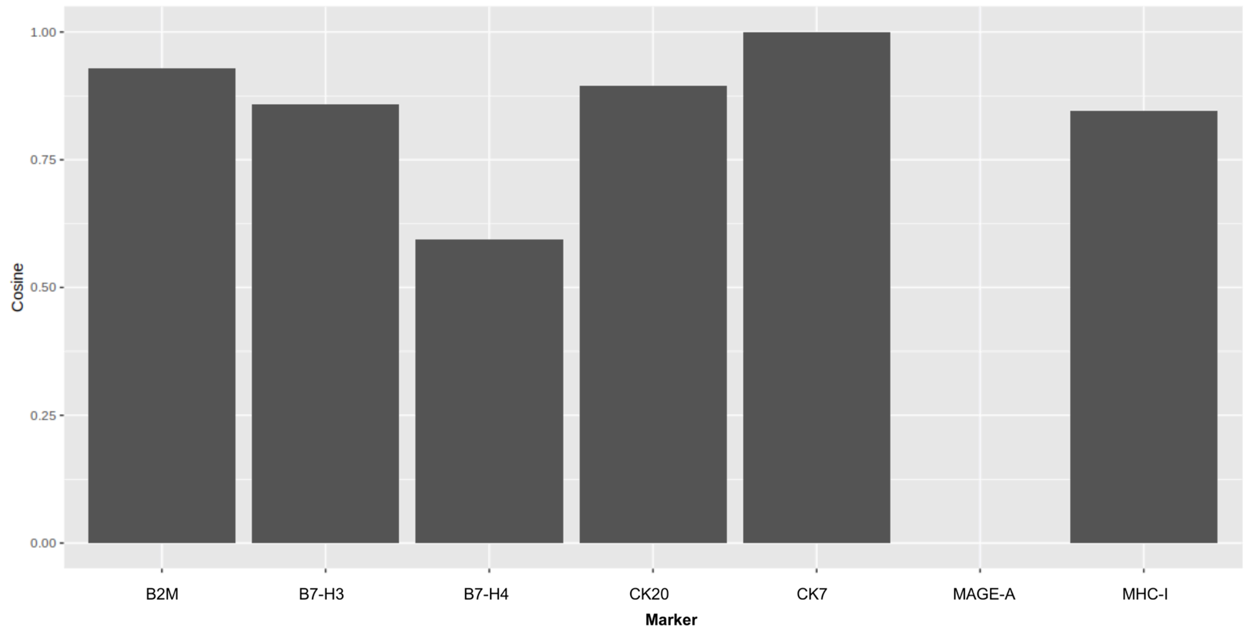

**Marker**	CK7	CK20	B7-H3	B7-H4	MAGE-A	B2M	MHC-I
**Cosine**	1.00	0.894	0.859	0.594	0.00	0.929	0.847

Supplementary Figure 9: Similarity between two resections from the same patient by marker. For each marker, the variation between an older resection (a) and a more recent resection (b) across all patients with two resections ($n = 6$) was measured according to cosine similarity. Most markers show at least a moderate degree of similarity, with CK7 and B2M showing little change between resection a and b. B7-H4 shows the least similarity between the multiple resections; most of the IHC scores were 0 for MAGE-A. PD-L1 is not shown as all patients with multiple resections had IHC scores equal to zero.
